# Luminescent Properties of (Ca_7_ZrAl_6_O_18_-Ca_3_Al_2_O_6_-CaZrO_3_):Eu^3+^ Composite Ceramics and Tracing in the Hydration Process

**DOI:** 10.3390/molecules28237799

**Published:** 2023-11-27

**Authors:** Dominika Madej, Andrzej Kruk

**Affiliations:** 1 Faculty of Materials Science and Ceramics, Department of Ceramics and Refractories, AGH University of Krakow, al. A. Mickiewicza 30, 30-059 Krakow, Poland; 2 Institute of Technology, University of the National Education Commission, Krakow, ul. Podchorążych 2, 30-084 Krakow, Poland

**Keywords:** Ca_7_ZrAl_6_O_18_:Eu ceramics, tracing the early and long-term hydration, structural and microstructural properties

## Abstract

In this work, solid-state reaction sintering was used to fabricate Ca_7_ZrAl_6_O_18_-Ca_3_Al_2_O_6_-CaZrO_3_:Eu^3+^ ternary composite ceramics and cements. The structural, microstructural, and spectroscopic properties of the ceramics with different Eu_2_O_3_ content were characterized by X-ray diffraction (XRD), Fourier-transform infrared spectroscopy (FT-IR), scanning electron microscopy (SEM) and energy dispersive X-ray spectroscopy (EDS), and spectrofluorimetry, respectively. The XRD patterns analyzed with Rietveld refinement confirm the presence of the orthorhombic phase of Ca_7_ZrAl_6_O_18_ and the cubic phase of Ca_3_Al_2_O_6_ in all the samples, indicating that doping of Eu^3+^ slightly changes the crystalline structure of both aluminate phosphors. EDS analysis revealed that the Eu doping element was strongly concentrated to the two phases, i.e., Ca_7_ZrAl_6_O_18_ and Ca_3_Al_2_O_6_, with the Eu concentrations of 8.45 wt.% and 8.26 wt.%, respectively. The luminescent properties of the ceramics doped with different Eu^3+^ ion concentrations were investigated by excitation and emission spectroscopy at room temperature. These results were compiled using a laser with an optical parametric oscillator (OPO) system. The obtained spectra indicated changes in the luminescence intensity and shape occurring with phase composition and Eu_2_O_3_ concentration. The emission spectra of the ceramics exhibit a strong dependence on the excitation wavelength in the range from 210 to 300 nm, and invariably, five peaks were assigned to the ^5^D_0_ → ^7^F_J_ (J = 0, 1, 2, 3, 4) transitions of Eu^3+^. The luminescence spectroscopy was then used to trace the early and long-term hydration behavior of cements. Thus, luminescence spectroscopy may provide a new tool for non-destructive testing of cement-based structures.

## 1. Introduction

In recent years, calcium zirconium aluminate (Ca_7_ZrAl_6_O_18_, C_7_A_3_Z; C = CaO, A = Al_2_O_3_, Z = ZrO_2_) has attracted much attention in the research and has been applied in materials science according to its hydrating and hardening abilities and its high-temperature stability due to the ZrO_2_ presence [1,2,3]. Ca_7_ZrAl_6_O_18_ belongs to the CaO-Al_2_O_3_-ZrO_2_ system together with the significant presence of other calcium aluminates—Ca_12_Al_14_O_33_ (C_12_A_7_), CaAl_2_O_4_ (CA), CaAl_4_O_7_ (CA_2_), and CaAl_12_O_19_ (CA_6_) [4,5]. Alkaline earth aluminates are low-cost metal oxides, widely used as suitable host lattices for trivalent rare-earth elements (REE^3+^:Pr^3+^, Sm^3+^, etc.) and divalent europium (Eu^2+^) ions for the preparation of light-emitting materials (phosphors) with persistent luminescence [6,7]. Both wet chemistry and solid-state reaction methods are commonly used to prepare these materials [8,9]. The group of metals referred to as REE comprises the 15 elements of the lanthanide series (Ln^3+^). In this regard, lanthanides have been used as substitutes for Ca^2+^ in calcium aluminate phases because of the similarities in their spherical shape, ionic radii, and high coordination numbers. The luminescent properties of calcium aluminates C_12_A_7_ [10,11], CA [12,13,14,15,16], CA_2_ [17,18], and CA_6_ [19,20] doped with Ln^3+^ have been intensively studied in recent years, but the role of C_7_A_3_Z in this matter is not known. Following this line of reasoning, it is to be expected that the Eu^3+^ ion can easily substitute Ca^2+^ in C_7_A_3_Z, as in other calcium aluminates. Thus, the chemical and structural properties of the Ca^2+^ ion sites could be probed by analyzing the spectroscopic response of the Eu^3+^ ions embedded in these sites in C_7_A_3_Z.

The latest developments and trends in cement chemistry research describe the Ln^3+^ ions, more specially, Eu^3+^ as an appealing choice in investigating the phase composition in hydrating cementitious systems [21,22,23]. Regarding this matter, the dynamics of Ordinary Portland Cement (OPC) hydration through luminescence and other correlated spectroscopy techniques was monitored by several authors [24,25]. For example, Santos et al. [24] used the photoluminescence spectroscopy in order to understand the OPC hydration within the first hours of hydration. The results confirmed a formation of the C-S-H (C = CaO, S = SiO_2_, H = H_2_O) phase initially after 1 min of hydration and a growing process up to 32 min. A study by Burek et al. [23] found that Time-Resolved Laser Fluorescence Spectroscopy (TRLFS) results using Eu^3+^ as an optical probe can provide information about the admixture influence on the course of the OPC hydration. The authors stated that the dip in the dependence of the luminescence decay times on the hydration time indicates the change in the structure of C-S-H in the early hydration period. In another study, Pointeau et al. [22] reported the sorption mechanisms of Eu^3+^ on the C-S-H phases of hydrated OPC as a tool for the prediction of the behavior of trivalent radionuclides with aged/degraded cements in radioactive waste repositories. This work confirmed that Eu^3+^ from the solution is sorbed on the C-S-H solid phase with fast kinetics and high efficiency within a few minutes of contact, confirming a possible high retention for analogous trivalent elements.

It is reasonable, therefore, to suppose that the hydration dynamics of calcium aluminate cements (CACs) and cements belonging to the CaO-Al_2_O_3_-ZrO_2_ system can also be monitored through Eu^3+^ emission luminescence spectroscopy, where Eu^3+^ occurs as a probe for the determination of the phase changes. The chemical, physical, and microstructural changes during cement hydration are complex and depend on time of hydration, temperature, water–cement ratio, chemical and phase compositions, admixtures, substituting ions in clinker phases, and interrelationships between phases [26,27]. Assessing these processes and recognizing the different hydrates belonging to the CaO-Al_2_O_3_-H_2_O system require special techniques such as X-ray Diffraction (XRD), Scanning Electron Microscopy/Energy Dispersive X-ray Spectrometry (SEM/EDS), Time-Resolved Electrochemical Impedance Spectroscopy (TR-EIS), microcalorimetry, Thermogravimetry (TG), Differential Thermal Analysis (DTA), Evolved Gas Analysis with MS detection (EGA-MS), etc. [28,29,30,31].

Recently, it was discovered that luminescence methods can be used to study the effect of hydration on the physicochemical properties of the rare-earth ions in OPC such as changes in chemical environment, sorption, or hydrolysis effects [21,22,24]. However, no investigations into the new (Ca_7_ZrAl_6_O_18_-Ca_3_Al_2_O_6_-CaZrO_3_):Eu ceramic composite with luminescent properties and its hydration behavior by luminescence spectroscopy have been reported. Hence, the main goal of this paper is to develop and implement a methodology of non-destructive testing of new aluminous cements belonging to the CaO-Al_2_O_3_-ZrO_2_ system via luminescence and excitation spectroscopic methods using Eu^3+^ as a structural probe. The proposed ceramics comprised three phases. Two phases are hydraulic—Ca_7_ZrAl_6_O_18_ and Ca_3_Al_2_O_6_—and a CaZrO_3_ phase has a high melting point. All three phase are interesting from the point of view of refractory cements that can be implemented in the monolithic refractory materials, especially castables. 

## 2. Results and Discussion

### 2.1. Phase Composition (XRD) and Microstructural Characterizations (SEM-EDS)

**Figure 1:**
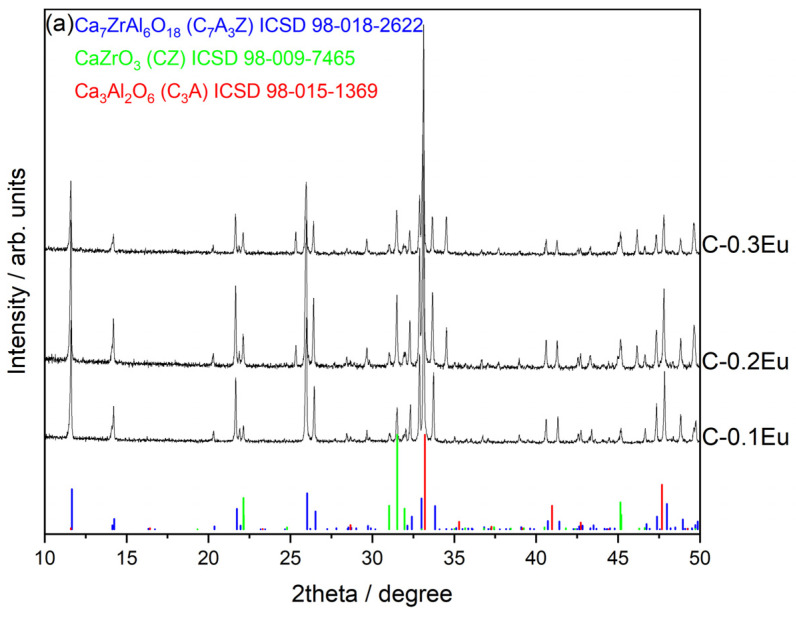

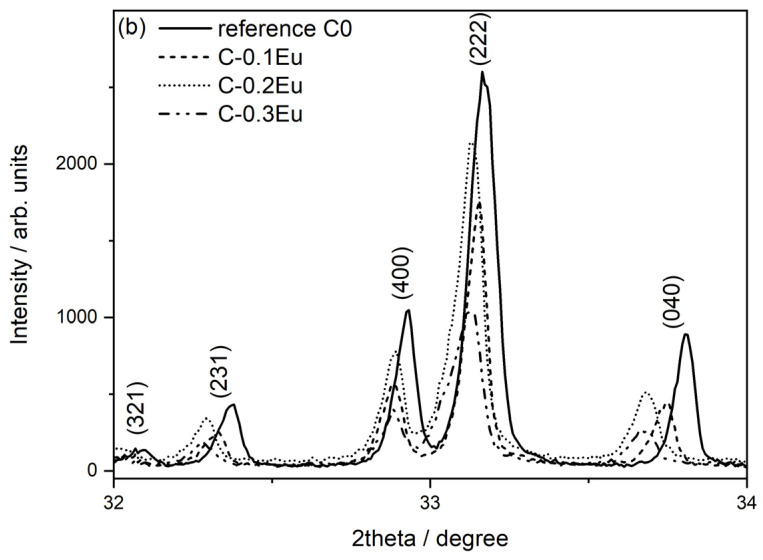
X-ray powder diffractograms of (**a**) the Eu-free reference C0, the Eu-containing C-0.1Eu, C-0.2Eu, and C-0.3Eu ceramics, and the reference peak positions of Ca_7_ZrAl_6_O_18_ (ICSD no. 98-018-2622), CaZrO_3_ (ICSD no. 98-009-7465), and Ca_3_Al_2_O_6_ (ICSD no. 98-015-1369) and of (**b**) the shift in X-ray diffraction peaks due to the lattice changes induced by Eu ion doping in Ca_7_ZrAl_6_O_18_ as a main phase.

To shed light upon the structural information and microstructure features of the multiphase C-0.1Eu, C-0.2Eu, and C-0.3Eu ceramics, X-ray diffraction (XRD) and scanning electron microscope (SEM) coupled with energy dispersive X-ray spectroscopy (EDS) were implemented. Figure 1a shows the XRD pattern of the multiphase powder mixtures. The vertical bars indicate peak positions from the powder diffraction standard cards of Ca_7_ZrAl_6_O_18_ (ICSD 98-018-2622), CaZrO_3_ (ICSD 98-009-7465), and Ca_3_Al_2_O_6_ (ICSD 98-015-1369). A fine scanning of the ceramics in the 2theta ranges 32–34° is shown in Figure 1b. All diffraction peaks can be indexed to the orthorhombic phase of calcium zirconium aluminate (Ca_7_ZrAl_6_O_18_) with Pmn21 space group (No. 31), the orthorhombic phase of calcium zirconium aluminate (CaZrO_3_) with Pnma space group (No. 62), and the cubic phase of tricalcium dialuminate (Ca_3_Al_2_O_6_) with Pm-3 m space group (No. 221), which are consistent with the ICSD PDF No. 98-018-2622, 98-009-7465, and ICSD 98-015-1369, respectively. No traces of additional peaks are observed in the XRD patterns, which confirm that the multiphase samples are made of calcium zirconium aluminate, calcium zirconate, and tricalcium dialuminate; the content of each phase deduced from Rietveld refinements is given in Table 1. In addition, there are no diffraction peaks of starting substrates or intermediate compounds in the sintered samples; therefore, it is inferred that they were reacted completely. As expected, the significant content of Eu_2_O_3_ affects the precipitation of two secondary phases (CaZrO_3_ and Ca_3_Al_2_O_6_) in all samples, and slight changes were observed in the unit cell parameters of Ca_7_ZrAl_6_O_18_ (Table 1) due to the similar ionic radii of Ca^2+^ (1.26 Å) and Eu^3+^ (1.206 Å) [12] cations. Similarity among ionic radii of calcium and europium ions makes the Ca_7_ZrAl_6_O_18_ a promising host lattice for the Eu^3+^ ion. In order to obtain more crystal structure information on Ca_7_ZrAl_6_O_18_ doped with Eu, the lattice parameters and theoretical density of the as-synthesized phase was calculated by the Rietveld refinement method using X’Pert HighScore Plus v3.0 software. The reliability index parameters—indicated as weighted profile R factor (R_wp_), unweighted profile R factor (R_p_), and expected R factor (R_exp_)—were estimated to be slightly below 10% and at a goodness-of-fit factor (GOF) of close to 1, ensuring the reliability of the XRD refinement result. Table 1 lists the calculated lattice parameters of Eu-doped Ca_7_ZrAl_6_O_18_ and the other admixture phases, together with those of the reported parameters for the undoped components. As Figure 1b confirms, the diffraction peak of the Ca_7_ZrAl_6_O_18_ shifted very slightly to a lower 2theta value in the sintered sample, indicating a small expansion to a higher lattice parameter (Table 1) because of the europium ions in the host substitute alkaline earth metals. 

**Figure 2:**
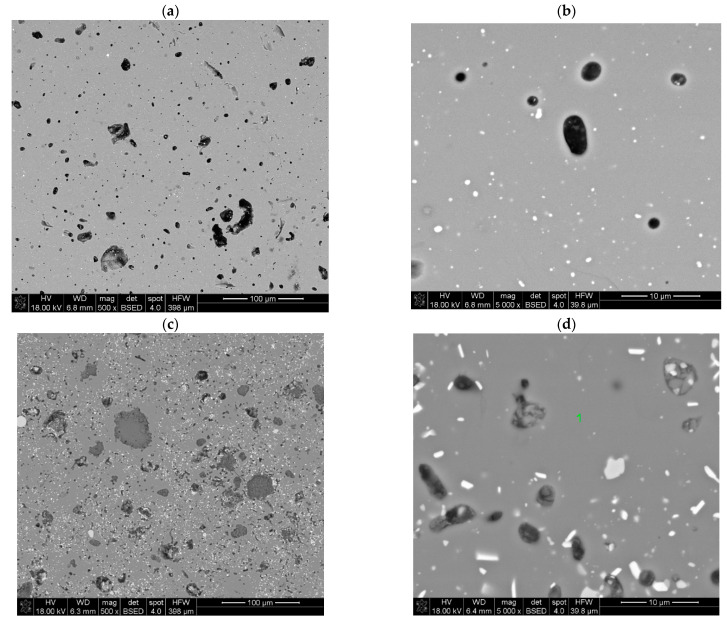

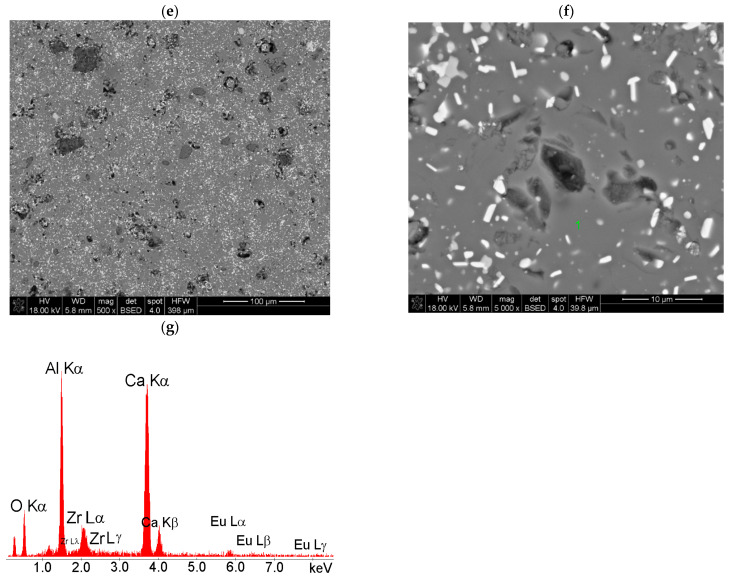
Scanning electron microscopy (SEM) images showing the microstructure of polished C-0.1Eu (**a**,**b**), C-0.2Eu (**c**,**d**), and C-0.3Eu (**e**,**f**) ceramics at different magnifications (Eu-doped Ca_7_ZrAl_6_O_18_ marked as a continuous gray matrix (marked as spot 1 in (**d**,**f**)), CaZrO_3_ marked as light gray inclusions, and Ca_3_Al_2_O_6_ marked as dark gray inclusions). (**g**) EDS spectrum of the gray matrix of C-0.3Eu ceramics shown in (**f**), showing the presence of calcium, aluminum, zirconium, oxygen, and europium.

Figure 2 exhibits the SEM images of the polished surfaces of the as-synthesized C-0.1Eu, C-0.2Eu, and C-0.3Eu ceramics. Typical three-phase samples can be revealed from Figure 2a–f. First of all, as indicated by XRD study results and the quantitative phase analysis using the Rietveld method of the as-synthesized C-0.1Eu, C-0.2Eu, and C-0.3Eu ceramics, it was proved that both CaZrO_3_ and Ca_3_Al_2_O_6_ occur as modifying agents and are present in small quantities (Table 1). The particles of CaZrO_3_ are visible in the SEM images as light gray areas, whereas Ca_3_Al_2_O_6_ occurs as dark gray areas in all samples (Figure 2a,c,e). The CaZrO_3_ as a secondary phase was nearly homogenously distributed in a gray matrix of Eu-doped Ca_7_ZrAl_6_O_18_, whereas Ca_3_Al_2_O_6_ formed a separated dark gray area. The X-ray spectrum of gray continuous phase indicates a composition of calcium, europium, aluminum and oxygen as the major elements in Eu-doped Ca_7_ZrAl_6_O_18_ solid solution grains (Figure 2g). It was also shown that with the increasing of Eu content in the starting blend powders, the concentration of Eu increases in all phases present in the sinters (Table 2, Table 3 and Table 4).

### 2.2. Structural Characterization (FT-IR)

**Figure 3:**
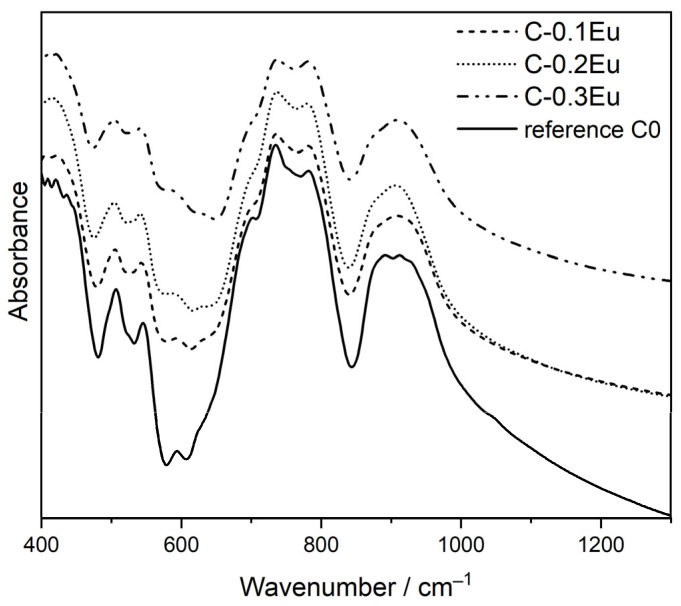
Fourier-transform infrared spectroscopy (FT-IR) of undoped reference C0 and Eu-containing C-0.1Eu, C-0.2Eu, and C-0.3Eu ceramics.

Fourier-transform infrared spectroscopy (FT-IR) is a simple and fast technique used for functional group identification in many inorganic materials, especially in cements and other cementitious materials [32]. Here, Figure 3 represents the FT-IR spectra of the synthesized reference C0 and the Eu-containing C-0.1Eu, C-0.2Eu, and C-0.3Eu ceramics. This figure shows that the FT-IR spectra of C-0.1Eu, C-0.2Eu, and C-0.3Eu ceramics are similar to each other and are similar to the undoped reference C0 sample. A consideration of all the FT-IR spectra found that Ca_7_ZrAl_6_O_18_ was the dominant phase in all Eu-containing samples. The FT-IR spectrum of Ca_7_ZrAl_6_O_18_ is noteworthy for very intense bands between 600 and 1000 cm^−1^ due to the vibrations of Al-O and of Ca-O bonds and for bands near 550 cm^−1^ due to the vibrations of Al-O [1]. Some small discrepancies between undoped reference material and Eu-containing samples may arise from the two admixtures of CaZrO_3_ and Ca_3_Al_2_O_6_ phases existing in the C-0.1Eu, C-0.2Eu, and C-0.3Eu ceramics. It is also worth mentioning that the Ca_7_ZrAl_6_O_18_ and Ca_3_Al_2_O_6_ have similar FT-IR spectra, indicating a similarity in the structure [33]. 

### 2.3. Particle Size Distribution of Eu-Containing Ceramic Powders

**Figure 4:**
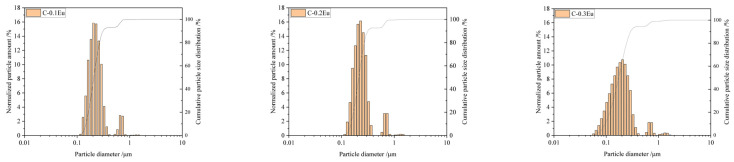
The particle size distribution (differential-bar graphs and cumulative-grey lines) for the three Eu-containing C-0.1Eu, C-0.2Eu, and C-0.3Eu ceramic powders.

The particle size distributions of Eu-containing ceramic powders are shown in Figure 4. The particle size distribution is nearly unimodal with a median diameter D50 of approximately 0.2 µm. The average particle size of powder is approximately 0.5 µm. The single particles show a tendency to aggregate (the minor peaks are approximately 0.8 µm). The control of the powder grain size enables selection of the hydration reaction rate and influences the luminescence [34,35]. The results clearly show that the C-0.3Eu powder has smaller particle sizes and a wider size distribution when a higher amount of Eu_2_O_3_ was used. In this case, the D50 is ca. 0.17 µm. The uniform sub-micro size of the powder is conducive to the formation of uniform fine grains, so as to improve the density and mechanical properties. 

### 2.4. Luminescent Properties of Eu-Containing Ceramic Powders 

**Figure 5:**
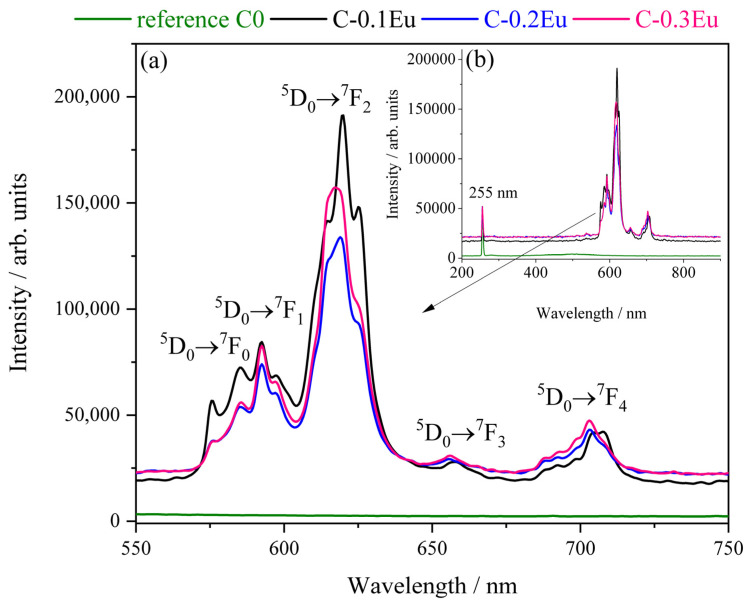
Luminescence intensity of the C0 reference and Eu-containing C-0.1Eu, C-0.2Eu, and C-0.3Eu ceramic powders excited at 255 nm (**a**). Inset figure (**b**) shows the full recorded spectrum including laser light.

Luminescence intensity of C-0.1Eu, C-0.2Eu, and C-0.3Eu ceramic powders has been found to depend on the wavelength excitation light and doping concentration. The hydration progress was monitored for the C-0.1Eu sample. For this purpose, luminescence was induced by coupling a laser with an OPO. The luminescence spectra of the reference undoped C0 and the Eu-containing C-0.1Eu, C-0.2Eu, and C-0.3Eu powders excited at a wavelength of 255 nm and recorded in the wavelength range of 550 and 750 nm at room temperature are presented in Figure 5. Figure 5 also compares the shape of the luminescence spectra for the C-0.1Eu, C-0.2Eu, and C-0.3Eu materials. The spectra are characterized by the wide emission from 560 to 720 nm, with the most prominent transition being ^5^D_0_ → ^7^F_2_ for all samples. For the undoped C0 powder, no luminescence was found in the investigated region (Figure 5b). The luminescence peaks for the C-0.1Eu powder are more intense than the luminescence peaks for the other powders after excitation at 255 nm (Figure 5b). It is also clearly visible that the C-0.1Eu spectrum is sharper and insignificantly wider. The PL emission spectra of C-0.2Eu and C-0.3Eu are similar due to the samples having a similar structure and phase composition. Moreover, Eu^3+^ ions were incorporated into the host lattice at a higher doping concentration in the C-0.3Eu sample (8.45 wt.% in Ca_7_ZrAl_6_O_18_ and 3.83 wt.% in Ca_3_Al_2_O_6_), and a prominent energy migration between the Eu^3+^ ions took place. This point can be related to the shorter distance of the Eu^3+^-Eu^3+^. As it was reported elsewhere [36,37], the luminescent properties of the alkaline earth aluminates depend on various factors including the nature, structure, and composition of the matrix, as well as the rare-earth ion content. As it was also reported elsewhere [38,39], both the high degree of similarity between the structures of the Ca_7_ZrAl_6_O_18_ and Ca_3_Al_2_O_6_ aluminates and the similarity among ionic radii of Ca^2+^ (1.26 Å) and Eu^3+^ (1.206 Å) make both aluminates promising host lattices for Eu^3+^ ions [12]. Going into details, the structure of Ca_3_Al_2_O_6_ consists of rings of six AlO_4_ tetrahedra ([Al_6_O_18_]) at eight to a unit cell, surrounding holes with Ca^2+^ ions holding the rings together. The Ca coordination polyhedra have marked departures from regular octahedra, but the AlO_4_ tetrahedra are much less distorted [38]. On the other hand, in the structure of Ca_7_ZrAl_6_O_18_, the five types of Ca atoms and four types of AlO_4_ tetrahedra were, respectively, positionally and orientationally disordered. In this structure, six AlO_4_ tetrahedra are joined, sharing corners to form [Al_6_O_18_] rings [39].

As shown in Figure 5a, the luminescence spectra of the three different Eu-containing powders have two distinct peaks in common at 593 and 620 nm, which were assigned to the magnetic dipole ^5^D_0_ → ^7^F_1_ and the electric dipole ^5^D_0_ → ^7^F_2_ transitions, respectively [22,40]. This magnetic dipole transition is forbidden by spin but allowed by the Laporte selection rule [41]. On the other hand, electronic transitions to *J* = 0,2,3,4 are strictly forbidden by selection rules purely for transitions when Eu^3+^ occupies a site with a strict center of symmetry. Nevertheless, emission spectra also include lines corresponding to transitions: ^5^D_0_ → ^7^F_0_ (ca. 580 nm), ^5^D_0_ → ^7^F_3_ (ca. 660 nm), and ^5^D_0_ → ^7^F_4_ (ca. 710 nm) [42]. Here, the Eu^3+^ must partially substitute the Ca^2+^ ions; it also occupies polyhedra sites in the host crystal structure of Ca_7_ZrAl_6_O_18_ and octahedral sites of Ca_3_Al_2_O_6_, contributing strong polyhedral distortion because all five transitions are visible in this spectra. The substitution of Ca^2+^ ions by Eu^3+^ ions is expected due to the similarities in their spherical shape, ionic radii, and high coordination numbers. Doping with Eu^3+^ proceeds with mostly substitution out of symmetry of a polyhedra site because red light is significantly stronger. So, the intensity of luminescence bands is related to the local symmetry of the Eu^3+^ activators rather than to the quantity of these ions. 

Interesting results were obtained by comparing the intensity ratio of ^5^D_0_ → ^7^F_1_ and ^5^D_0_ → ^7^F_2_ transition bands for the studied powders. The intensities of the magnetic transitions that occur in Eu^3+^ ions are quite similar for all the investigated samples; however, the electric dipole ^5^D_0_ → ^7^F_2_ is significantly different in intensities. The differences between the emission spectra of the C-0.1Eu sample and the two other C-0.2Eu and C-0.3Eu samples likely originate, firstly, from the content of both Ca_7_ZrAl_6_O_18_ and Ca_3_Al_2_O_6_ and, secondly, from the concentration of Eu^3+^ ions in these phases (Table 2, Table 3 and Table 4). Moreover, since the europium ion probes the symmetry disorder in the crystal structure of Eu-containing samples, the changes of the chemical environment of Eu^3+^ in hydrating systems remains interesting, especially in terms of explaining the still unclear kinetics of cement hydration at the very early stage. This point will be further discussed. This intensity ratio can be further used as a parameter needed for estimating the “degree of asymmetry” of a crystal site.

**Figure 6:**
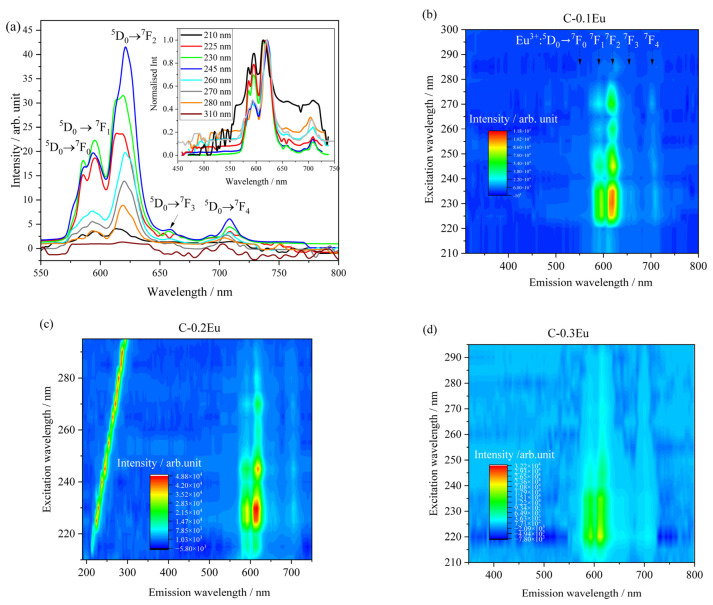
(**a**) Luminescence spectra of the C-0.2Eu powder excited by different wavelengths. (**b**–**d**) Excitation–emission contour plots for C-0.1Eu, C-0.2Eu, and C-0.3Eu powders. The color scale shows emission intensity in arbitrary units.

The evolution of luminescence shape and intensity as a function of the excitation light is presented in Figure 6. The selected luminescence spectra of C-0.2Eu powder are shown in Figure 6a, as a representative. In this study, excitation lights were also normalized. In fact, the overlapping ^5^D_0_ → ^7^F_0_ band can be observed after excitation of selected wavelengths. Also, the wide ^5^D_0_ → ^7^F_2_ band consisting of two peaks is clearly observed. The main ^5^D_0_ → ^7^F_2_ band significantly increases with the increase in energy of the laser light up to ~240 nm (5.1 eV). In this peak, the two components are denoted as S_1_ and S_2_. Generally, the S_1_ is higher for all chemical compositions when the powder is excited by higher wavelengths. The area under the ^5^D_0_ → ^7^F_4_ band shows quantitatively that intensity changes the least for different laser wavelengths.

The evolution of PL emission as a function of laser wavelength for C-0.1Eu, C-0.2Eu, and C-0.3Eu powders differing in Eu_2_0_3_ content and phase composition is presented as the excitation–emission map in Figure 6b–d. The intensity of emitting light increases with the increasing wavelength of the light up to ca. 230 nm. Above this wavelength value, the intensity of the luminescence decreases until it disappears completely above and beyond 300 nm. The intensity of the peaks centered at approximately ca. 617 nm is the highest for each sample, whereas the luminescence spectral ratios of 593 nm and 617 nm are clearly different. The excitation lights are shown as an example in Figure 6c.

**Figure 7:**
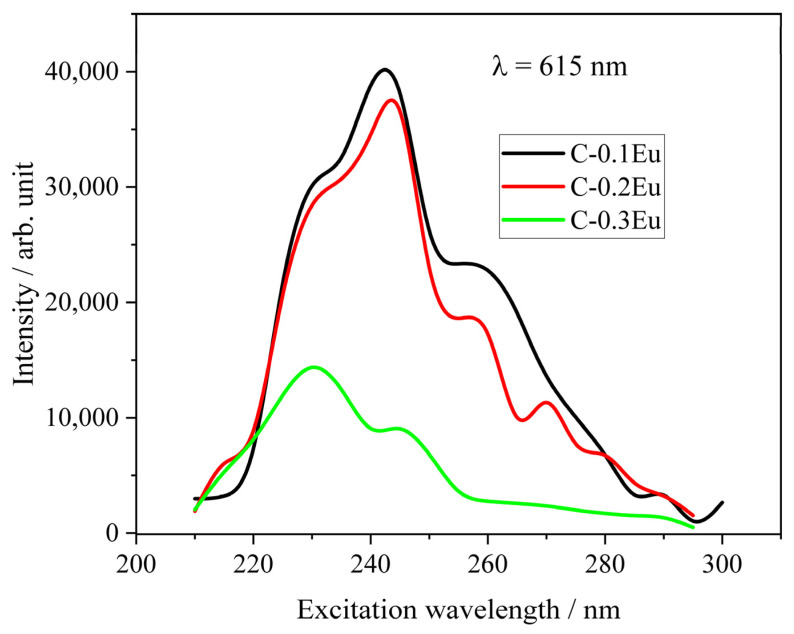
Excitation spectrum for emission at 615 nm measured for the powders.

For detailed studies, the excitation spectra of the powders monitored at 615 nm corresponding to the ^5^D_0_ → ^7^F_2_ transition were displayed in Figure 7. The excitation spectra showed only one broad band ranging from 570 to 725 nm corresponding to the Eu^3+^ (Figure 7). It was found that the excitation spectral features are quite similar in shape for all cement powders with the maximum centered at 245 nm (Figure 7). The luminescence light increases with an increase in the pump wavelength up to 245 nm due to an increase in photons absorption. When the wavelength of peak absorption reaches a maximum, the luminescence continuously decreases. The excitation spectrum of C-0.1Eu and C-0.2Eu are similar to the absorption spectrum of Eu^3+^ [43]. The excitation spectrum for C-0.3Eu powder at a shorter wavelength is comparable to that for Eu-O emission [44]. This means that the Eu-O emission corresponds to the Eu_2_O_3_-rich phase. In the case of this powder, the quenching of luminescence is observed due to the concentrated quenching of luminescence in the Eu^3+^-containing sample.

### 2.5. Tracing in the Hydration Process of Eu-Containing Ceramic Powder via Luminescence and XRD Techniques

In the next stage of this work, the hydration process of Eu-containing cement at the very early stage was investigated using Eu^3+^ as an optical probe. For this purpose, the C-0.1Eu sample was taken. These results illustrate the utility of Eu^3+^ probes to signal changes in the local chemical environment of hydrating cement particles, especially the dissolution and precipitation of new solids. It was previously investigated and discussed that the hydration of Ca_7_ZrAl_6_O_18_ as a main phase (and Ca_3_Al_2_O_6_ as a secondary phase) proceeds, firstly, through the dissolution of initial cement particles and the formation of metastable phases belonging to the CaO-Al_2_O_3_-H_2_O system and, secondly, through the formation of stable hydrates [1,2,3,4,5,39]. Furthermore, the main hydration products are the gel-like and quasi-amorphous calcium aluminate hydrates at the very early stage formed immediately after the dissolution.

**Figure 8:**
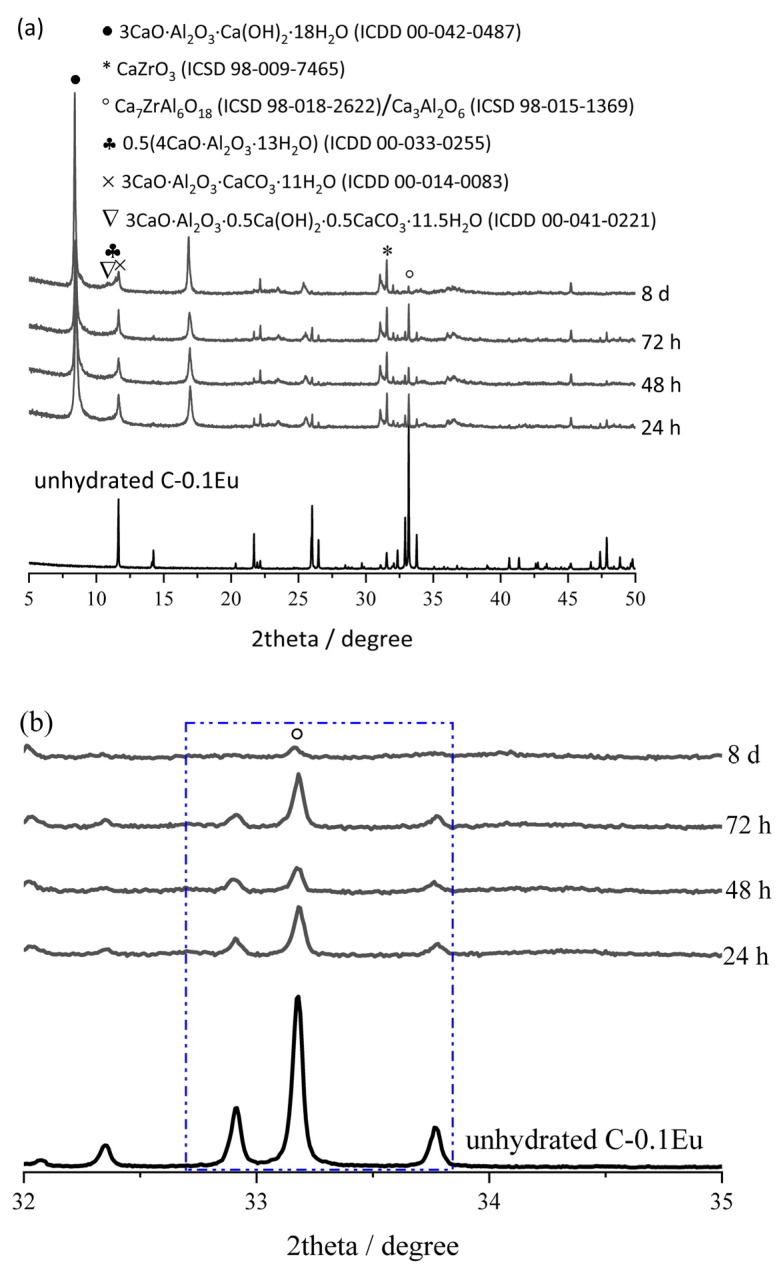
X-ray diffractograms of C-0.1Eu cement unhydrated and hydrated between 24 h and 8 days (**a**). XRD profiles of the main diffraction peak positions belonging to Ca_7_ZrAl_6_O_18_ (and Ca_3_Al_2_O_6_ as minor phase) in the cement pastes hydrated between 24 h and 8 days (**b**). The blue box indicates the main peaks belonging to Ca_7_ZrAl_6_O_18_.

In order to point out the phase evolution with the time of the chemical reaction between components of the C-0.1Eu sample and water, X-ray diffraction studies were implemented. The results of X-ray diffractogram evaluation show the formation of various crystalline phases after 24, 48, 72 h, and 8 days of curing, which are more or less the same for all hydrated samples. The X-ray diffractograms of the hydrated samples are shown in Figure 8a along with the X-ray diffractogram of unhydrated cement. The main diffraction peaks corresponding to the major compound in the unhydrated cement (Figure 8a) were of calcium zirconium aluminate (Ca_7_ZrAl_6_O_18_; ICSD 98-018-2622) and minor tricalcium aluminate (Ca_3_Al_2_O_6_; ICSD 98-015-1369) at 33.213° 2θ (CuKα), and calcium zirconate (CaZrO_3_; ICSD 98-009-7465) at 31.513° 2θ (CuKα). The XRD profiles of the main diffraction peak position belonging to Ca_7_ZrAl_6_O_18_ and Ca_3_Al_2_O_6_ are undistinguishable due to their similar d-spacing (d = 2.69527Å in Ca_7_ZrAl_6_O_18_ and d = 2.69549 Å in Ca_3_Al_2_O_6_). The intensity of the peak at 33.213 2θ (CuKα) of Ca_7_ZrAl_6_O_18_ decreased in hydrated pastes due to the hydration and formation of calcium aluminate hydrates (xCaO·yAl_2_O_3_·zH_2_O) (Figure 8b). The C_3_A·Ca(OH)_2_·18H_2_O hydrate (ICDD 00-042-0487) was identified at 2θ (CuKα) of 8.301° and 16.651° (Figure 8a). The peaks of the 0.5(4CaO·Al_2_O_3_·13H_2_O) hydrate (ICDD 00-033-0255) were identified at 2θ (CuKα) of 11.263° and 31.138°. The peak of the 3CaO·Al_2_O_3_·CaCO_3_·11H_2_O hydrate (ICDD 00-014-0083) was also observed at 11.681°, and the 3CaO·Al_2_O_3_·0.5Ca(OH)_2_·0.5CaCO_3_·11.5H_2_O hydrate (ICDD 00-041-0221) peak was observed at 10.781°.

**Figure 9:**
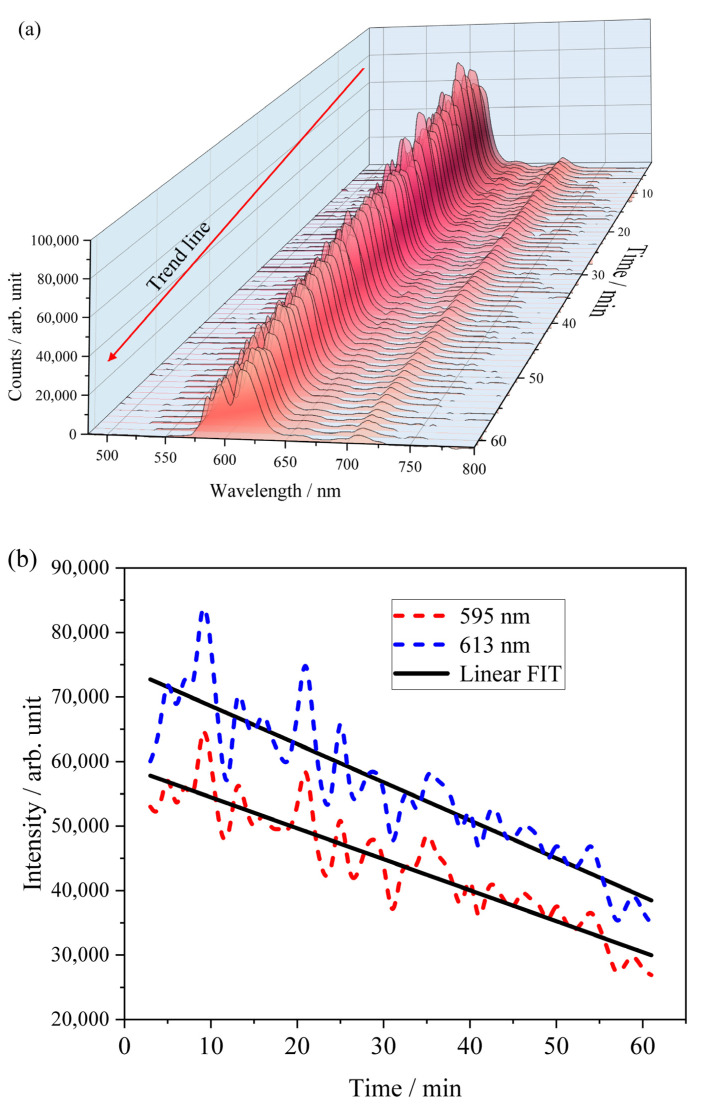
Time evolution of luminescence spectra of the C-0.1Eu-H fresh paste excited by a 230 nm wavelength.

The time evolution of the luminescence intensity of C-0.1Eu-H paste (powder + water, H = H_2_O) is shown in Figure 9a. Analysis of the emission spectra has demonstrated that the intensity and shape strongly depend on time. The luminescence emission spectra are dominated by the ^5^D_0_ → ^7^F_2_ band emission peaking at 397 nm. A plot of change in luminescence intensity at 617 nm recorded for the C-0.1Eu-H paste as a function of the hydration time process is shown in Figure 9b. Straight lines were fitted to the experimental data with a fairly good accuracy. The corresponding maximum intensities of emission spectra are arranged along straight lines as shown in Figure 9b. The slopes of straight lines were estimated as −480 and −607 for emission peaks at 595 nm and 613 nm, respectively. It suggests that the electric dipole transitions of the europium ions ^5^D_0_ → ^7^F_2_ decrease faster than those of the magnetic dipole ^5^D_0_ → ^7^F_1_ transitions. Two or more variables are considered to be related in this context. A first variable can be connected with the quenching effect of water to reduce the luminescence of europium ions, and a second one can be due to changes in the structural disorder.

**Figure 10:**
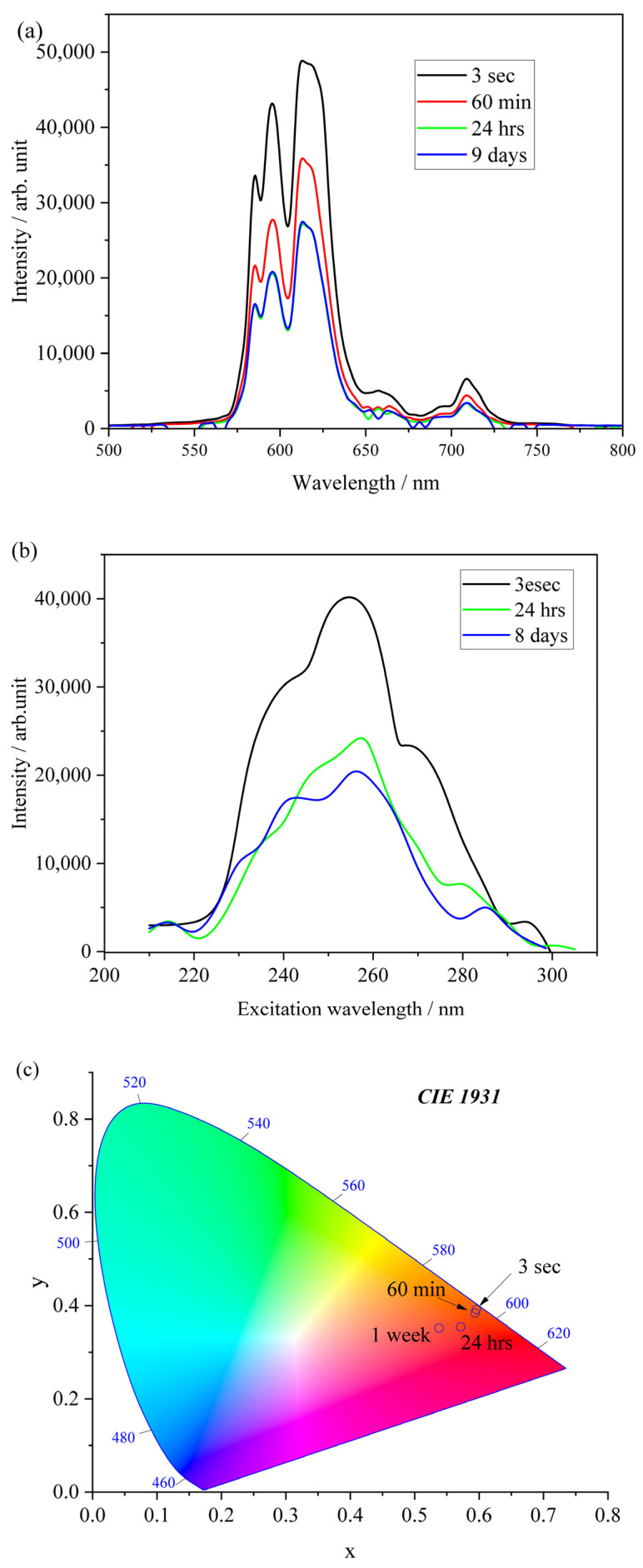
The long-term dependence of Eu luminescence spectra in hydrating C-0.1Eu-H material excited at 250 nm of pump wavelength (**a**); excitation spectra in hydrating C-0.1Eu-H material monitored at 615 nm (**b**); and CIE chromaticity coordinates for hydrating C-0.1Eu-H material (**c**).

In the deep analysis of the spectra at a very early state of cement hydration (Figure 9a), Eu^3+^ ions may be released and may concentrate in the cement pore solution, leading to a high quenching concentration and being physically bound to the rigid gel of poorly crystalline CaO-Al_2_O_3_-H_2_O hydrates in the next stage of hydration. Nevertheless, the resultant spectrum is thus a superposition of many component spectra including unhydrated particles and intermediate hydrates. The hydration of both Eu-doped Ca_7_ZrAl_6_O_18_ and Ca_3_Al_2_O_6_ appears to be significantly influenced by time in the long-term curing process (Figure 10a–c). The long-term luminescence studies revealed that the emission band intensity did not change significantly, which means that the Eu^3+^ ions have stable positions and could be physically or chemically bound to the crystalline CaO-Al_2_O_3_-H_2_O hydrates, as confirmed by XRD. Preceding hydration processes did not change the distance between Eu^3+^ ions; therefore, the interactions between Eu^3+^ ions are the same, including cross-relaxation energy transfer. The CIE color coordinates of hydrated paste in different times are shown in Figure 10c. This evolution of luminescence is confirmed by the change in color of the paste, in which a shift from red to white light emission is observed with time. This color change is related to the change in the ratio of asymmetry.

Excitation spectra are observed at the ^5^D_0_ → ^7^F_2_ band emission peaking at 615 nm. This material shows a continuous decrease in the luminescence intensity with hydration time. The shapes are quite similar and do not show a significant change. With the progress of hydration, it can be seen that the material shows a higher luminescence for higher excitation energies.

## 3. Materials and Methods

### 3.1. Synthesis of Eu-Containing Composite Ceramics, Cement Powders, and Methods of Investigation

The state-of-the-art Eu-containing Ca_7_ZrAl_6_O_18_-Ca_3_Al_2_O_6_-CaZrO_3_ composite ceramics were prepared using a two-step firing procedure based on the low-temperature calcination and high-temperature sintering processes. The starting materials utilized were CaCO_3_ (POCH, 99.9%), Al_2_O_3_ (Acros Organics, 99.0%), ZrO_2_ (Acros Organics, 98.5%), and Eu_2_O_3_ (Acros Organics, 99.99%). In all the experiments, the raw materials were first dried at 110 °C for 5 h due to their hygroscopic nature. The designed series of the new class of ceramics (Table 5) were synthesized by a solid-state reaction in air. The stoichiometric amounts of the raw materials were weighed and dry mixed in a zirconium ball mill for 2 h. Then, the well-mixed starting reactants were pressed into cylinders with a diameter of 20 mm and height of 10 mm. All samples were subsequently calcined at 1300 °C for 10 h in air, well grounded in an agate mortar, milled in a zirconium ball mill for 2 h again, and then pressed and sintered at 1400 °C for 15 h in air. The reference C0 ceramics were sintered at 1420 °C for 15 h in air. Repeated grindings were performed between the calcination and sintering processes to improve the mixing homogeneity.

The microstructure of a series of as-synthesized Eu-containing ceramics was analyzed by scanning electron microscopy (SEM, NOVA NANO SEM 200 of FEI) and equipped with an EDS system of EDAX to determine the microstructural features, especially phase distribution. To this purpose, resin-embedded sinters were ground, polished, covered by a thin layer of conductive material, and finally examined. The sinters were then crushed and milled until particle size decreased below 63 μm. Powder X-ray diffraction (XRD, X-ray diffractometer from PANalytical-Empyrean model) with a Cu Kα X-ray source (λ = 0.154187 nm, 2θ = 10–50°) was performed to detect the crystal phases of the sintered samples. The FT-IR spectra of Eu-containing ceramics were recorded in the range of 1400–400 cm^−1^ using the KBr pellets technique on a BRUKER (Vertex 70) Fourier-Transform Infrared Spectrometer. The Shimadzu SALD-7500nano was used to characterize the particle size distribution of three—C-0.1Eu, C-0.2Eu, and C-0.3Eu—ceramic powders dissolved in methanol by utilizing the static light scattering (SLS) technique. The equipment has a semiconductor laser (405 nm) and a reverse Fourier optical system.

The luminescence spectra of the powders were detected from a StellarNet SilverNova spectrometer (with resolution 0.5 nm) using a tunable laser (Ekspla, Vilnius, Lithuania) as the excitation (laser with an optical parametric oscillator (OPO) system) source. The spectra were collected by 906 ms. The output light has 30–50 mJ per pulse, in the wavelength range from 210 to 300 nm, with a typical pulse duration of 4–5 ns. The excitation lights from the laser were normalized to the maximum intensity because the OPO generates a single pulse with slightly different power. The neutral density filters placed in the output of the laser beam make it possible to adjust the energy of the laser beam. To guarantee the reproducibility of the data in this study, the sample was placed in a precision positioning system. The detection of luminescence light was conducted through an optical fiber SMA—type (Fiber_UV_50, Spectra-Laser) with low UV light absorption. The optical collimator was not used due to high luminescence effects. The emission spectra of the powders were measured at room temperature. Two types of measurements were performed: emission spectra, in which the intensity is recorded as a function of laser beams of different wavelengths, and excitation spectra, in which the intensity of the selected wavelength is recorded as a function of the excitation wavelength.

### 3.2. Cement Paste Preparation and Methods of Investigation

As time progresses, the initial flash formation of the XRD-undetected calcium aluminate hydrate coatings around the grains of Ca_7_ZrAl_6_O_18_ and Ca_3_Al_2_O_6_ hydraulic phases and the growth from the amorphous phase of crystalline phases between 0.5 and 1 h take place [1,2,45]. In order to explore the influence of hydration development of Eu-containing cements on their luminescent performance, the luminescence behavior of Eu-containing specimens at different times of hydration was measured and compared. For this purpose, tests for both short- and long-term effects of hydration on luminescent properties were performed. We used the same optical set-up and methodology for determination of the luminescent properties of the cement pastes as described in Section 2.1. Here, three types of measurements were performed: emission spectra, excitation spectra, and an additional emission spectra, in which the intensity is recorded as a function of time for one selected laser beam wavelength.

The composition of the tested powder was C-0.1Eu. The fresh cement paste (powder + water) abbreviated as C-0.1Eu-H was prepared at a constant water to cement mass ratio (w/c) of 0.5 and an ambient temperature of 21 °C. The cement paste was strongly mixed by hand to reach a homogenous paste. Then, tests for the short-term effect of hydration on luminescent properties were performed immediately, and the luminescence spectra were collected over a period of 1 h with 1 min intervals. Then, the hydrated sample was cured at 85% relative humidity at 21 °C for 8 days after casting. The long-term effects of hydration on luminescent properties were examined at 24 h and at 8 days of storing. The XRD method was applied to analyze the hydration progress of C-0.1Eu-H paste at 24, 48, and 72 h and at 8 days of curing. At the end of each step of hydration, acetone was used for the interruption of material hydration.

## 4. Conclusions

Eu-containing ceramic materials composed of three phases were successfully prepared by a solid-state reaction in an oxidizing atmosphere. Synthesized samples were characterized structurally, microstructurally, and spectroscopically using XRD, FT-IR, SEM-EDS, and spectrofluorimetry, respectively. X-ray diffraction and Rietveld refinement studies of the samples confirm the formation of Ca_7_ZrAl_6_O_18_, along with a small concentration of Ca_3_Al_2_O_6_ (0.1–1.6 wt.%) and CaZrO_3_ (4.9–8.9 wt.%). From the XRD analysis, an orthorhombic phase of Ca_7_ZrAl_6_O_18_ and a cubic phase of Ca_3_Al_2_O_6_ were reported in all the samples, indicating that the doping of Eu^3+^ slightly changes the crystalline structure of both calcium zirconium aluminate and tricalcium aluminate phosphors. Thus, the identification and synthesis of Eu-doped Ca_7_ZrAl_6_O_18_ as a new potential phosphorus were done. Eu-containing ceramic materials were efficiently excited by the deep ultraviolet light (210–300 nm) and showed a wide-range red emission. In the Eu-containing ceramics sintered at 1400 °C, transitions of Eu^3+^ for four electric dipoles ^5^D_0_ → ^7^F_0, 2–4_ and one magnetic dipole ^5^D_0_ → ^7^F_1_ appear as wide peaks with high intensity. Variations in the luminescence of three Eu-containing ceramics were attributed to a positional change of europium polyhedra and local bond distortion in the unit cell of both Ca_7_ZrAl_6_O_18_ and Ca_3_Al_2_O_6_ phases. The results show that the increase in Eu_2_O_3_ concentration can reduce both the luminescence intensities and the excitation intensity of Eu^3+^ due to the energy transfers between Eu^3+^-Eu^3+^.

Furthermore, the possibility to optically excite Eu^3+^ in the hydrating cement matrix opens the way to their application in intelligent monitoring and assessment of early-age hydration and the setting of cements. The luminescence spectra of the Eu-containing cement paste (powder + water) at a very early stage of hydration exhibited five decreasing distinct peaks, with two maxima at 593 and 620 nm, corresponding to the magnetic dipole ^5^D_0_ → ^7^F_1_ and the electric dipole ^5^D_0_ → ^7^F_2_ transitions, respectively. The corresponding maximum intensities of emission spectra decreased linearly with different negative slopes due to the dissolution of initial cement particles and the formation of metastable phases belonging to the CaO-Al_2_O_3_-H_2_O system. We also employed excitation spectroscopy to understand the long-term effects of hydration on luminescent properties of cements because this method has excellent sensitivity in compliance with the emission intensity. The results of this study suggest that the luminescence color of hydration products can be used as an obvious qualitative feature to trace the hydration process of Ca_7_ZrAl_6_O_18_-Ca_3_Al_2_O_6_:Eu. It was, for the first time, possible to get insights into the hydration of calcium aluminates using luminescence spectroscopy. In the near future, the combination of XRD, SEM, FT-IR, and luminescence spectroscopy might become a key tool for the refractory aluminous cement industry.

## Figures and Tables

**Table 1:** Rietveld quantitative phase analysis results and refined lattice parameters of the phases formed in the C-0.1Eu, C-0.2Eu, and C-0.3Eu ceramics with relative errors.

Sample	Rietveld quantitative phase analysis, wt.%	**Phase**	**Unit Cell Parameters/** **Å**		
			**a**	**b**	**c**
		Ca_7_ZrAl_6_O_18_ (ICSD PDF No. 98-018-2622)	10.8490	10.5910	7.6690
		CaZrO_3_ (ICSD PDF No. 98-009-7465)	5.7610	8.0200	5.5940
		Ca_3_Al_2_O_6_ (ICSD PDF No. 98-015-1369)	7.6240	7.6240	7.6240
C-0.1Eu	95.0	Ca_7_ZrAl_6_O_18_	10.87890 ± 0.00275	10.6100 ± 0.00179	7.6698 ± 0.0008
	4.9	CaZrO_3_	5.7489 ± 0.0021	8.0159 ± 0.0005	5.5954 ± 0.00025
	0.1	Ca_3_Al_2_O_6_	7.1995 ± 0.0556	7.1995 ± 0.00025	7.1995 ± 0.00025
C-0.2Eu	92.2	Ca_7_ZrAl_6_O_18_	10.8761 ± 0.00249	10.6265 ± 0.00335	7.6709 ± 0.00025
	7.1	CaZrO_3_	5.7527 ± 0.00144	8.0159 ± 0.0005	5.5961 ± 0.00037
	0.7	Ca_3_Al_2_O_6_	8.2747 ± 0.0853	8.2747 ± 0.0853	8.2747 ± 0.0853
C-0.3Eu	89.5	Ca_7_ZrAl_6_O_18_	10.8745 ± 0.0023	10.6265 ± 0.0033	7.6705 ± 0.0061
	8.9	CaZrO_3_	5.7522 ± 0.00153	8.0173 ± 0.00034	5.5971 ± 0.00055
	1.6	Ca_3_Al_2_O_6_	7.3354 ± 0.0393	7.3354 ± 0.03785	7.3354 ± 0.03785

**Table 2:** EDS analysis of the Eu-doped Ca_7_ZrAl_6_O_18_ phase (gray continuous phase) together with those of reference Ca_7_ZrAl_6_O_18_ given as an average of five points.

Sample	Elements in wt.%				
	Ca	Eu	Al	Zr	O
Theoretical composition of pure Ca_7_ZrAl_6_O_18_	34.17	-	19.65	11.11	35.07
C0	33.85	-	23.86	11.38	30.91
C-0.1Eu	33.30	3.29	19.36	11.01	33.04
C-0.2Eu	32.70	5.21	18.70	10.95	32.44
C-0.3Eu	31.85	8.45	19.49	10.97	29.24

**Table 3:** EDS analysis of the Eu-doped Ca_3_Al_2_O_6_ phase (dark gray inclusions) together with those of reference Ca_3_Al_2_O_6_ given as an average of five points.

Sample	Elements in wt.%			
	Ca	Eu	Al	O
Theoretical composition of pure Ca_3_Al_2_O_6_	44.50	-	19.97	35.53
C0	42.89	-	20.65	36.46
C-0.1Eu	41.82	2.54	19.27	36.37
C-0.2Eu	40.76	3.55	19.58	36.11
C-0.3Eu	40.19	3.83	19.86	36.12

**Table 4:** EDS analysis of the Eu-doped CaZrO_3_ phase (light gray inclusions) together with those of reference CaZrO_3_ given as an average of five points.

Sample	Elements in wt.%			
	Ca	Eu	Zr	O
Theoretical composition of pure CaZrO_3_	22.35	-	50.88	26.77
C0	20.51	-	48.13	31.36
C-0.1Eu	20.07	1.82	47.14	30.97
C-0.2Eu	19.50	2.60	47.62	30.28
C-0.3Eu	19.08	3.21	46.80	30.91

**Table 5:** Composition of Eu-containing ceramics.

Sample Code	Composite Type
C0	Ca_7_ZrAl_6_O_18_
C-0.1Eu *	(Ca_7_ZrAl_6_O_18_-Ca_3_Al_2_O_6_-CaZrO_3_):0.1Eu^3+^
C-0.2Eu *	(Ca_7_ZrAl_6_O_18_-Ca_3_Al_2_O_6_-CaZrO_3_):0.2Eu^3+^
C-0.3Eu *	(Ca_7_ZrAl_6_O_18_-Ca_3_Al_2_O_6_-CaZrO_3_):0.3Eu^3+^

* 0.1Eu = 0.1Eu_2_O_3_, 0.2Eu = 0.2Eu_2_O_3_, 0.3Eu = 0.3Eu_2_O_3_.

## Data Availability

Data are contained within the article.
